# Efficacy of three neuroprotective drugs in secondary progressive multiple sclerosis (MS-SMART): a phase 2b, multiarm, double-blind, randomised placebo-controlled trial

**DOI:** 10.1016/S1474-4422(19)30485-5

**Published:** 2020-03

**Authors:** Jeremy Chataway, Floriana De Angelis, Peter Connick, Richard A Parker, Domenico Plantone, Anisha Doshi, Nevin John, Jonathan Stutters, David MacManus, Ferran Prados Carrasco, Frederik Barkhof, Sebastien Ourselin, Marie Braisher, Moira Ross, Gina Cranswick, Sue H Pavitt, Gavin Giovannoni, Claudia Angela Gandini Wheeler-Kingshott, Clive Hawkins, Basil Sharrack, Roger Bastow, Christopher J Weir, Nigel Stallard, Siddharthan Chandran, MS-SMART Investigators, Jeremy Chataway, Claudia A.M. Gandini Wheeler-Kingshott, Floriana De Angelis, Domenico Plantone, Anisha Doshi, Nevin John, Thomas Williams, Marie Braisher, Tiggy Beyene, Vanessa Bassan, Alvin Zapata, Siddharthan Chandran, Peter Connick, Dawn Lyle, James Cameron, Daisy Mollison, Shuna Colville, Baljean Dhillon, Christopher J. Weir, Richard A. Parker, Moira Ross, Gina Cranswick, Gavin Giovannoni, Sharmilee Gnanapavan, Richard Nicholas, Waqar Rashid, Julia Aram, Helen Ford, James Overell, Carolyn Young, Heinke Arndt, Martin Duddy, Joe Guadagno, Nikolaos Evangelou, Matthew Craner, Jacqueline Palace, Jeremy Hobart, Basil Sharrack, David Paling, Clive Hawkins, Seema Kalra, Brendan McLean, Nigel Stallard, Roger Bastow

**Affiliations:** a Queen Square Multiple Sclerosis Centre, Department of Neuroinflammation, University College London (UCL) Queen Square Institute of Neurology, Faculty of Brain Sciences, UCL, London, UK; b Department of Medical Physics and Biomedical Engineering, Centre for Medical Image Computing, UCL, London, UK; c Centre for Clinical Brain Sciences, University of Edinburgh, Edinburgh, UK; d Edinburgh Clinical Trials Unit, Usher Institute, University of Edinburgh, Edinburgh, UK; e Universitat Oberta de Catalunya, Barcelona, Spain; f Department of Radiology and Nuclear Medicine, VU University Medical Centre, Amsterdam, Netherlands; g School of Biomedical Engineering & Imaging Sciences, King's College London, London, UK; h Dental Translational and Clinical Research Unit, University of Leeds, Leeds, UK; i Blizard Institute, Barts and The London School of Medicine and Dentistry, Queen Mary University, London, UK; j Brain MRI 3T Research Center, IRCCS Mondino Foundation, Pavia, Italy; k Keele Medical School and Institute for Science and Technology in Medicine, Keele University, Keele, UK; l Department of Neuroscience, Royal Hallamshire Hospital, Sheffield, UK; m Patient Representative, Multiple Sclerosis Society, London, UK; n Statistics and Epidemiology, Division of Health Sciences, Warwick Medical School, University of Warwick, Coventry, UK; o National Institute for Health Research, University College London Hospitals, Biomedical Research Centre, London, UK

## Abstract

**Background:**

Neurodegeneration is the pathological substrate that causes major disability in secondary progressive multiple sclerosis. A synthesis of preclinical and clinical research identified three neuroprotective drugs acting on different axonal pathobiologies. We aimed to test the efficacy of these drugs in an efficient manner with respect to time, cost, and patient resource.

**Methods:**

We did a phase 2b, multiarm, parallel group, double-blind, randomised placebo-controlled trial at 13 clinical neuroscience centres in the UK. We recruited patients (aged 25–65 years) with secondary progressive multiple sclerosis who were not on disease-modifying treatment and who had an Expanded Disability Status Scale (EDSS) score of 4·0–6·5. Participants were randomly assigned (1:1:1:1) at baseline, by a research nurse using a centralised web-based service, to receive twice-daily oral treatment of either amiloride 5 mg, fluoxetine 20 mg, riluzole 50 mg, or placebo for 96 weeks. The randomisation procedure included minimisation based on sex, age, EDSS score at randomisation, and trial site. Capsules were identical in appearance to achieve masking. Patients, investigators, and MRI readers were unaware of treatment allocation. The primary outcome measure was volumetric MRI percentage brain volume change (PBVC) from baseline to 96 weeks, analysed using multiple regression, adjusting for baseline normalised brain volume and minimisation criteria. The primary analysis was a complete-case analysis based on the intention-to-treat population (all patients with data at week 96). This trial is registered with ClinicalTrials.gov, NCT01910259.

**Findings:**

Between Jan 29, 2015, and June 22, 2016, 445 patients were randomly allocated amiloride (n=111), fluoxetine (n=111), riluzole (n=111), or placebo (n=112). The primary analysis included 393 patients who were allocated amiloride (n=99), fluoxetine (n=96), riluzole (n=99), and placebo (n=99). No difference was noted between any active treatment and placebo in PBVC (amiloride *vs* placebo, 0·0% [95% CI −0·4 to 0·5; p=0·99]; fluoxetine *vs* placebo −0·1% [–0·5 to 0·3; p=0·86]; riluzole *vs* placebo −0·1% [–0·6 to 0·3; p=0·77]). No emergent safety issues were reported. The incidence of serious adverse events was low and similar across study groups (ten [9%] patients in the amiloride group, seven [6%] in the fluoxetine group, 12 [11%] in the riluzole group, and 13 [12%] in the placebo group). The most common serious adverse events were infections and infestations. Three patients died during the study, from causes judged unrelated to active treatment; one patient assigned amiloride died from metastatic lung cancer, one patient assigned riluzole died from ischaemic heart disease and coronary artery thrombosis, and one patient assigned fluoxetine had a sudden death (primary cause) with multiple sclerosis and obesity listed as secondary causes.

**Interpretation:**

The absence of evidence for neuroprotection in this adequately powered trial indicates that exclusively targeting these aspects of axonal pathobiology in patients with secondary progressive multiple sclerosis is insufficient to mitigate neuroaxonal loss. These findings argue for investigation of different mechanistic targets and future consideration of combination treatment trials. This trial provides a template for future simultaneous testing of multiple disease-modifying medicines in neurological medicine.

**Funding:**

Efficacy and Mechanism Evaluation (EME) Programme, an MRC and NIHR partnership, UK Multiple Sclerosis Society, and US National Multiple Sclerosis Society.

## Introduction

Multiple sclerosis includes both inflammatory and neurodegenerative pathological mechanisms in the CNS. Neurodegenerative features form the dominant substrate of progressive multiple sclerosis and manifest clinically by irreversible accumulation of disability.^1^ Progressive multiple sclerosis is the major cause of disease-associated costs, both to individuals and health care systems^2^ and, therefore, it is a key target for therapeutic development. However, by contrast with the range of treatments that mitigate inflammatory activity in relapsing-remitting multiple sclerosis, treatments that can slow, stop, or reverse progressive multiple sclerosis are limited.

**Research in context**

**Evidence before this study**

We have previously published a systematic review and synthesis of available evidence for candidate oral neuroprotective drugs tested in clinical trials from patients with multiple sclerosis, dementia, motor neuron disease, Huntington's disease, and Parkinson's disease, combined with in-vivo data from experimental autoimmune encephalomyelitis (EAE) studies. We did two further searches in Ovid MEDLINE and Epub Ahead of Print, In-Process & Other Non-Indexed Citations and Daily (from 1946 to Feb 27, 2019), OVID Embase (from 1980 to 2019 week 8), the Cochrane Database of Systematic Reviews, and the Cochrane Central Register of Controlled Trials (CENTRAL). In the first search, we used a combination of keywords and database-appropriate subject headings for the trial drugs: “amiloride” OR “fluoxetine” OR “riluzole” AND “multiple sclerosis” OR “experimental allergic encephalomyelitis” OR “EAE”. We excluded symptomatic human studies. We did not restrict our search by language. In the second search, we used a combination of keywords and database-appropriate subject headings for neurodegenerative neurological diseases, including “multiple sclerosis” OR “Parkinson's disease” OR “amyotrophic lateral sclerosis-motor neuron disease” OR “Huntingdon's disease” OR “dementia”, combined with terms to retrieve multiarm drug trials and the Cochrane Highly Sensitive Search Strategy for identifying randomised trials in MEDLINE (sensitivity and precision-maximising version, 2008 revision). We excluded symptomatic, dose-ranging, non-drug trials or studies of relapsing-remitting multiple sclerosis. We did not restrict our search by language. The first search retrieved five experimental studies for amiloride, indicating potential neuroprotection in animal models and human pathological samples. The most likely mechanism was blockage of ASIC1. In a clinical trial of primary progressive multiple sclerosis (n=14), a significant reduction was noted in the rate of whole-brain volume change with amiloride as well as improvements in deep grey and white matter tract diffusion indices. In three EAE studies, fluoxetine partly ameliorated paralysis and reduced inflammatory foci. Findings of two MR spectroscopy studies in humans showed an increase in N-acetylaspartate variables with fluoxetine, suggesting improved neuronal energetics and microstructural integrity by diffusion MRI, although a third study showed no change in markers of phosphocreatine metabolism. In a small placebo-controlled study, patients with relapsing multiple sclerosis treated with fluoxetine showed a trend towards a reduction in the number of new enhancing lesions over time. In a pilot study in progressive multiple sclerosis (n=42), non-significant benefits were seen in some markers of clinical progression with fluoxetine, although the study was underpowered. Riluzole reduced the severity of inflammation, demyelination, and axonal damage in a myelin oligodendrocyte glycoprotein-induced EAE system. In a run-in study in 16 patients with primary progressive multiple sclerosis, a reduction was noted in the rate of cervical atrophy and new T1 hypointense lesions with riluzole. In another study, no effect was seen on the atrophy rate in early multiple sclerosis with riluzole. The second search for multiarm drug trials in neurodegenerative diseases retrieved three studies. The first study in patients with progressive multiple sclerosis (n=58) assessed cyclophosphamide, adrenocorticotropic hormone, and plasma exchange. In the second study, 782 patients with early Parkinson's disease were randomly allocated to approved treatments (levodopa and DOPA decarboxylase inhibitor alone; levodopa, DOPA decarboxylase inhibitor, and selegiline; or bromocriptine). The third study was a three-arm trial of tianzhi granule, donepezil, or placebo in improving functional ability in vascular dementia. No other multiarm trials were retrieved by our search.

**Added value of this study**

In the MS-SMART trial, we chose three agents (amiloride, fluoxetine, and riluzole) to target different mechanistic pathways in patients with secondary progressive multiple sclerosis. We used a multiarm design to enable simultaneous assessment of these drugs at the important phase 2b decision point and, therefore, to accelerate the drug discovery process. The study design was robust with appropriate performance characteristics. We did not find any evidence of a neuroprotective effect of the three agents, despite positive early work in animals and humans.

**Implications of all the available evidence**

Multiarm trials are feasible and efficient in neurodegenerative diseases. They have the potential to examine promising experimental and early-phase agents in a timely fashion. The results of our study have implications for future experimental paradigms. This style of approach is necessary to accelerate treatment discovery in an area in which limited progress has been made.

The diverse pathobiological mechanisms that contribute to neuroaxonal loss in progressive multiple sclerosis provide a range of potential targets. Important proof-of-concept findings have emerged for drugs that have anti-inflammatory mechanisms of action, such as siponimod^3^ and ocrelizumab.^4^ In parallel, evidence has been accumulating for a range of candidate therapies that target key molecular processes in the axon itself, representing a more direct or downstream approach to achieve neuroprotection. Over the past three decades, many negative trials have been published, with the findings attributable in part to suboptimum trial design.^5^ However, the fundamental question of whether direct targeting of axonal pathobiological features can be an effective therapeutic strategy remains unanswered.

A further issue for adequate testing of an axonal-targeted neuroprotective strategy in people with progressive multiple sclerosis is the predictive value of drug selection from a large array of candidates. We did a systematic review and synthesis of available evidence for candidate oral neuroprotective drugs tested in clinical trials from patients with multiple sclerosis, dementia, motor neuron disease, Huntington's disease, or Parkinson's disease, combined with in-vivo data from experimental allergic encephalitis (EAE) studies.^6^ This approach resulted in a shortlist of seven candidate drugs with different mechanisms of action that might prove beneficial in progressive multiple sclerosis: ibudilast, oxcarbazepine, pirfenidone, polyunsaturated fatty acids (including lipoic acid), amiloride, fluoxetine, and riluzole. This strategy was ultimately confirmed by positive phase 2 results for two of the drugs, ibudilast^7^ and lipoic acid.^8^

Based on our previous work,^6^ we aimed to test the efficacy of targeting axonal pathobiological features as a strategy to achieve neuroprotection in progressive multiple sclerosis. To maximise efficiency of testing and accelerate drug development, we used a multiarm approach that enabled simultaneous assessment of several drugs at the important phase 2b decision point, a strategy that has been used successfully in oncology.^9^ Based on efficacy and drug supply, we selected three drugs from our candidate shortlist (amiloride, fluoxetine, and riluzole) for assessment against placebo.

Amiloride, widely used as a potassium-sparing diuretic, is an acid-sensing ion channel blocker.^10^, ^11^ ASIC1 opens in response to inflammation-induced acidosis, causing sodium and calcium influxes.^10^ This action is associated with axonal injury in post-mortem studies of patients with acute multiple sclerosis,^11^ and blockade of ASIC1 with amiloride reduces axonal damage and improves clinical outcomes in rodent models.^10^ A pilot study in individuals with progressive multiple sclerosis showed a significant reduction in whole-brain atrophy.^12^

Fluoxetine, a selective serotonin reuptake inhibitor used for depression, has pleiotropic neuroprotective effects stimulating glycogenolysis and improving mitochondrial energy metabolism.^13^, ^14^ In an underpowered negative trial, fluoxetine showed non-significant benefits in some markers of clinical progression.^15^

Riluzole, licenced for motor neuron disease, reduces glutamate release and antagonises voltage-dependent sodium channels.^16^ Glutamate excitotoxicity results in neuronal injury^17^ and its blockade in EAE reduces clinical impairment and axonal damage.^18^, ^19^, ^20^ In a pilot study of riluzole in people with progressive multiple sclerosis, a reduction in the rate of cervical cord atrophy and the number of new brain T1 hypointense lesions was recorded,^21^ although findings of another study in individuals with early relapsing-remitting multiple sclerosis or clinical isolated syndrome did not show a reduction in the rate of atrophy.^22^

The Multiple Sclerosis-Secondary Progressive Multi-Arm Randomisation Trial (MS-SMART) is a phase 2b, multicentre, multiarm, parallel group, double-blind, randomised placebo-controlled trial. We used the rate of brain atrophy to assess the putative neuroprotective effect of amiloride, fluoxetine, and riluzole in people with secondary progressive multiple sclerosis.^23^ Our aim was to efficiently test the efficacy of targeting axonal pathobiology as a strategy to achieve neuroprotection in progressive multiple sclerosis.

## Methods

### Study design and participants

We did an investigator-led, multiarm, parallel group, double-blind, randomised placebo-controlled trial of amiloride, fluoxetine, or riluzole versus placebo at 13 neuroscience centres in the UK. We screened patients for enrolment and included those aged 25–65 years with a diagnosis of secondary progressive multiple sclerosis, confirmed as per usual clinical practice.^24^, ^25^, ^26^

Major inclusion criteria were an Expanded Disability Status Scale (EDSS) score between 4·0 and 6·5, evidence of steady disability progression in the preceding 2 years (with either an increase of at least 1 point in EDSS score or a clinically documented increase in disability), and no concurrent use of disease-modifying therapies (standard UK practice for patients with secondary progressive multiple sclerosis). Patients were ineligible for the study if they had primary progressive multiple sclerosis, significant depression (Beck's Depression Index II score >19), major comorbidity, glaucoma, or epilepsy; were not able to undertake MRI; had a relapse or had been treated with corticosteroids within 3 months of screening; or used immunosuppressants, disease-modifying treatments, or experimental drugs within the previous 6 or 12 months (depending on the agent). Further details on the protocol, eligibility criteria, and study design are available elsewhere^23^ and in the appendix (pp 2–3).

The study was done in accordance with the Declaration of Helsinki and International Council for Harmonisation Good Clinical Practice guidelines. Independent ethics approval for the protocol was granted by REC 13/SS/0007, and all patients provided written informed consent before entering the study. Safety oversight was the responsibility of the Data Monitoring Committee, which reviewed accruing participant-level data every 6 months. Individual site medical monitoring was also mandated.

### Randomisation and masking

Within 30 days after screening for enrolment to the study, we randomly allocated patients (1:1:1:1) at baseline either amiloride, fluoxetine, riluzole, or placebo. A research nurse used a centralised web-based service provided by the Edinburgh Clinical Trials Unit (Usher Institute, University of Edinburgh, Edinburgh, UK) to randomly assign interventions, with minimisation by sex, age (<45 years *vs* ≥45 years), EDSS score at randomisation (4·0–5·5 *vs* 6·0–6·5), and trial site. The minimisation procedure incorporated a random element whereby the assigned treatment was switched with a probability of 10% from the group that would give greatest balance to one of the other three study groups (with a probability of 3·33% for each of the other study groups). Amiloride, fluoxetine, riluzole, and placebo capsules were over-encapsulated and identical in appearance. Patients and investigators, including MRI analysts, treating clinicians, and independent assessing neurologists were unaware of treatment allocations and had no access to randomisation codes. We asked patients and clinicians to complete a questionnaire at week 96 to assess the validity of the masking procedures.

### Procedures

We initially administered assigned treatments orally once daily, from baseline (week 0) for 4 weeks, then patients received doses twice daily from week 4 until week 96. Doses were amiloride hydrochloride 5 mg, fluoxetine 20 mg, riluzole 50 mg, or matching placebo. After the baseline visit, patients were seen at weeks 4, 8, 12, 24, 36, 48, 72, and 96, with a final safety telephone call at week 100. Brain MRI was done at screening for enrolment, week 24, and week 96. Neurological assessments were done at screening, baseline, week 48, and week 96. Safety blood tests were assessed at every study visit and included full blood count, electrolytes, and liver and renal function tests. Study treatment was discontinued if confirmed repeat measurements showed the following blood test concentrations: potassium less than 2·8 mmol/L or more than 5·5 mmol/L; sodium less than 125 mmol/L; alanine aminotransferase, aspartate aminotransferase, or γ-glutamyl transferase more than five times the upper limit of normal; creatinine more than 130 μmol/L; neutrophil count less than 1·0 × 10^9^ cells per L, platelet count less than 50 × 10^9^ cells per L; or haemoglobin less than 80 g/L. Adverse events were assessed at every study visit.

### Outcomes

The primary endpoint was the percentage brain volume change (PBVC) between baseline and 96 weeks, which is the standard primary outcome in phase 2 trials in progressive multiple sclerosis. We used the Structural Image Evaluation using Normalization of Atrophy (SIENA) method.^27^, ^28^ SIENA is an automated method that registers the follow-up scan to the baseline scan and produces an integral of the edge motion occurring in each voxel between scans and directly calculates the PBVC from those values.

MRI secondary endpoints were counts of new or enlarging T2 lesions at 96 weeks and PBVC at 24 weeks. The core MRI protocol included fluid-attenuated inversion recovery (FLAIR), proton density, and T2 and volumetric T1-weighted scans, as previously described.^23^ MRI data were analysed independently at a central reading site (Queen Square Multiple Sclerosis Centre, University College London, London, UK). Clinical secondary endpoints were changes from baseline to weeks 48 and 96 in EDSS score, the Timed 25-Foot Walk, the 9-Hole Peg Test, the Paced Auditory Serial Addition Test, the Multiple Sclerosis Functional Composite score, the Symbol Digit Modalities Test, high contrast (100%) visual acuity, and Sloan low contrast visual acuity (contrast 5%, 2·5%, and 1·25%).

Time to first relapse was recorded. Patient-reported outcomes were also measured at baseline, week 48, and week 96 using: the Multiple Sclerosis Impact Scale 29 items version 2; the Multiple Sclerosis Walking Scale version 2; the Neurological Fatigue Index; and health-related quality-of-life (measured with EuroQol five dimensions five levels [EQ-5D-5L]). Neuropathic pain scores were also obtained and will be reported separately. The Multiple Sclerosis Functional Composite Z-score was normalised (signed square-root transformed) using participants' baseline scores.

### Statistical analysis

The sample size calculation for the trial was based on a study from Altmann and colleagues.^29^ The percentage of expected total cohort dropouts was based on two phase 2 studies in secondary progressive multiple sclerosis,^30^, ^31^ from which we calculated that including 110 patients per study group would provide 90% statistical power in the analysis of covariance to detect a 40% reduction in PBVC versus placebo, allowing for 10% dropouts plus 10% of participants discontinuing treatment while remaining in follow-up.

Baseline data were described by summary statistics. A multiple regression model was fitted to the PBVC outcome variable, with study group as an explanatory factor (using placebo as the reference category), adjusting for baseline normalised brain volume and the minimisation variables (age, sex, treatment centre, and EDSS score at randomisation). The multicentre trial design was taken into account by adjusting for treatment centre as a fixed effect. For each pairwise comparison of active treatment versus control, we calculated the mean difference in PBVC. Additionally, we used the method of Dunnett to adjust 95% simultaneous CIs,^32^ to allow for multiple pairwise comparisons to a common control group and maintain the overall family-wise error rate below 5%. Dunnett-adjusted p values are reported for the primary outcome analyses.^33^, ^34^ The primary analysis was a complete-case analysis based on the intention-to-treat population. The intention-to-treat population included all patients in the MS-SMART trial who underwent randomisation and had data available at week 96.

The effects of missing data or outliers on the primary outcome findings were investigated by doing three sensitivity analyses. These entailed excluding outliers more than 4 SD away from the mean, imputing missing data using a standard multiple imputation method, and imputing missing data under a missing-not-at-random assumption, whereby a constant value was added to the values imputed using multiple imputation equal to the observed SD in the primary outcome at 96 weeks.

A further primary outcome analysis was done in the per-protocol population, which included participants who were adherent to the protocol and compliant with the originally assigned treatment throughout the duration of follow-up. Patients were judged compliant with the assigned treatment if they reported taking, on average, 90% or more of their prescribed medication (taking account of planned down-titrations and deferred up-titrations) in the 30 days preceding every clinic visit.

Secondary outcome analyses were not adjusted for multiple comparisons and used 5% as the nominal significance level. The number of new or enlarging T2 lesions detected at the 96-week MRI scan was compared between each of the three active treatment groups and placebo by means of an overdispersed Poisson regression model fitted to the number of new or enlarging T2 lesions at 96 weeks, with adjustment for minimisation variables.

For continuous or ordinal outcomes measured at 96 weeks (ie, the EDSS score, the 9-Hole Peg Test, the Paced Auditory Serial Addition Test, the Multiple Sclerosis Functional Composite score, the Symbol Digit Modalities Test, Sloan low contrast visual acuity, the Multiple Sclerosis Impact Scale 29 items version 2, the Multiple Sclerosis Walking Scale version 2, the Neurological Fatigue Index, and health-related quality of life), we used a multiple linear regression method to calculate adjusted mean differences and 95% CIs for the individual comparisons between each active treatment and placebo. Regression models were adjusted for baseline and the minimisation variables.

For the EDSS outcome only, 95% CIs were calculated using a bootstrap method due to the ordinal nature of the outcome variable. Cox proportional-hazard models adjusting for the minimisation variables were used for time-to-first relapse and the Timed 25-Foot Walk test at 96 weeks. Analyses were done using SAS version 9.4 (SAS Institute, Cary, NC, USA).

This trial is registered with ClinicalTrials.gov, NCT01910259.

### Role of the funding source

The funder had no role in study design, data collection, data analysis, data interpretation, or writing of the report. The corresponding author had full access to all data in the study and had final responsibility for the decision to submit for publication.

## Results

**Figure 1:**
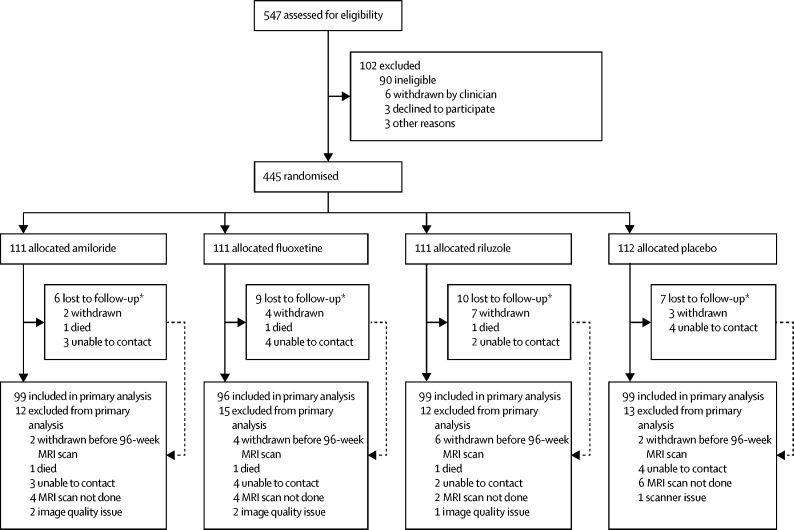
Trial profile *All patients lost to follow-up at any time during active follow-up (ie, up to and including the 100-week telephone call). Two patients withdrew after the 96-week MRI scan but before the end of the study (one allocated riluzole and one allocated placebo) and were included in the primary analysis. Two patients allocated riluzole also received fluoxetine prescribed by their family doctor towards the end of the trial. One patient allocated riluzole was withdrawn by a clinician: all other withdrawals were the patient's decision.

The study began in December, 2014, and ended in July, 2018. We screened 547 individuals for enrolment to the study, of whom 102 were judged ineligible or declined to participate (figure 1). Between Jan 29, 2015, and June 22, 2016, we randomly allocated 445 patients with secondary progressive multiple sclerosis either amiloride (n=111), fluoxetine (n=111), riluzole (n=111), or placebo (n=112). Baseline demographic characteristics were comparable between study groups, including whole-brain and T2 lesion volumes (table 1). The percentage loss to follow-up was 5–9% across study groups. Primary outcome data at 96 weeks were available for 393 (88%) of 445 participants, 99 of whom were assigned amiloride, 96 fluoxetine, 99 riluzole, and 99 placebo.

**Table 1:** Baseline characteristics of randomised participants

		**Amiloride (n=111)**	**Fluoxetine (n=111)**	**Riluzole (n=111)**	**Placebo (n=112)**	**Total (n=445)**
Age (years)		55·2 (49·0–60·5)	55·5 (50·7–60·2)	55·1 (49·7–59·2)	56·4 (49·2–60·4)	55·5 (49·7–60·3)
Male sex		36 (32%)	37 (33%)	37 (33%)	37 (33%)	147 (33%)
Female sex		75 (68%)	74 (67%)	74 (67%)	75 (67%)	298 (67%)
Expanded Disability Status Scale score		6·0 (5·5–6·5)	6·0 (5·5–6·5)	6·0 (5·5–6·5)	6·0 (5·5–6·5)	6·0 (5·5–6·5)
Time since first symptoms (years)		20 (13–30)	21 (16–29)	21 (16–26)	19 (13–29)	21 (15–29)
Time since progression (years)		6 (4–11)	5 (3–10)	6 (4–10)	5 (3–10)	6 (3–10)
Beck Depression Index II score		6 (4–9)	6 (3–10)	7 (4–12)	7 (4–12)	6 (4–11)
Multiple Sclerosis Functional Composite score (Z-score)		−0·19 (1·19)	−0·02 (0·60)	−0·09 (0·95)	−0·00 (0·91)	−0·07 (0·93)
Paced Auditory Serial Addition Test		39·0 (13·7)	36·6 (15·2)	36·9 (16·0)	41·5 (13·9)	38·5 (14·8)
Timed 25-Foot Walk (s)		12·0 (8·0–23·0)	11·0 (8·5–18·0)	11·4 (8·6–18·4)	10·6 (7·8–15·0)	11·2 (8·4–18·6)
9-Hole Peg Test (s^−1^)		0·03 (0·01)	0·03 (0·01)	0·03 (0·01)	0·03 (0·01)	0·03 (0·01)
Symbol Digit Modalities Test		43·9 (12·4)	44·1 (11·4)	44·5 (13·1)	44·1 (12·8)	44·2 (12·4)
High contrast (100%) visual acuity		50·1 (11·2)	50·8 (10·8)	48·5 (14·8)	50·4 (12·7)	49·9 (12·5)
Sloan low contrast visual acuity						
	5% contrast	32·6 (13·4)	32·9 (12·8)	30·0 (16·1)	33·9 (14·6)	32·4 (14·3)
	2·5% contrast	19·1 (12·7)	17·6 (12·4)	18·8 (14·2)	20·8 (14·0)	19·1 (13·3)
	1·25% contrast	8·2 (10·7)	6·9 (9·6)	7·1 (10·7)	9·9 (11·9)	8·0 (10·8)
Multiple Sclerosis Impact Scale 29 items version 2						
	Total score	63·9 (13·4)	65·0 (13·8)	69·2 (15·0)	66·1 (14·4)	66·0 (14·3)
	Physical score	48·0 (10·5)	48·3 (10·4)	51·0 (11·3)	49·0 (11·2)	49·1 (10·9)
	Psychological score	15·9 (4·5)	16·7 (4·8)	18·2 (5·4)	17·1 (5·0)	17·0 (5·0)
Multiple Sclerosis Walking Scale version 2		41·4 (9·2)	41·1 (9·8)	42·6 (9·3)	41·6 (9·9)	41·7 (9·5)
Neurological Fatigue Index						
	Summary score	18·0 (4·2)	17·4 (3·9)	19·1 (4·8)	17·8 (3·9)	18·1 (4·2)
	Physical score	15·1 (3·8)	14·7 (3·9)	15·9 (4·3)	14·7 (3·7)	15·1 (3·9)
	Cognitive score	6·5 (2·2)	6·2 (2·2)	7·1 (2·3)	6·3 (2·4)	6·5 (2·3)
	Diurnal score	10·1 (2·8)	9·6 (2·9)	10·2 (3·1)	9·7 (2·5)	9·9 (2·8)
	Nocturnal score	7·7 (2·0)	7·6 (2·5)	8·2 (2·8)	8·0 (2·3)	7·9 (2·4)
EQ-5D-5L						
	Index score	0·68 (0·17)	0·70 (0·16)	0·66 (0·17)	0·67 (0·18)	0·68 (0·17)
	VAS score	66·1 (16·9)	67·5 (19·5)	61·7 (21·0)	65·2 (20·3)	65·2 (19·5)
At least one relapse in past 2 years		15 (14%)	9 (8%)	6 (5%)	12 (11%)	42 (9%)
Baseline normalised brain volume (mL)		1432·2 (84·2)	1413·1 (82·4)	1414·2 (74·8)	1431·0 (91·1)	1422·6 (83·6)
T2 lesion volume (mL)		10·1 (3·5–17·4)	10·7 (4·8–20·4)	10·5 (3·8–19·6)	10·6 (4·4–17·6)	10·4 (4·1–18·6)

Data presented are n (%), mean (SD), or median (IQR). No data were missing for the 9-Hole Peg Test, Multiple Sclerosis Impact Scale 29 items version 2, and EQ-5D-5L (VAS score); for the remaining variables, varying amounts of data were missing up to a maximum of 12 patients overall (maximum five for amiloride, two for fluoxetine, three for riluzole, and four for placebo). EQ-5D-5L=EuroQol five dimensions five levels. VAS=visual analogue scale.

**Figure 2:**
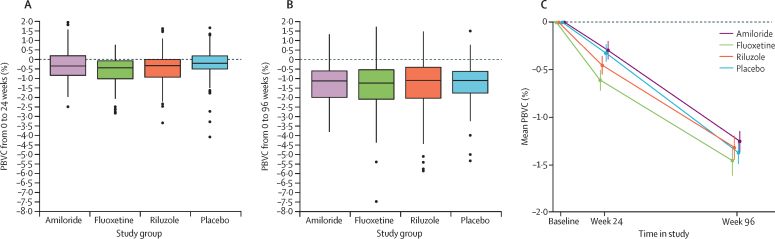
Primary outcome Boxplots of PBVC by study group at 24 weeks (A) and 96 weeks (B). Horizontal lines are median and IQR; whiskers extend to the minimum and maximum within 1·5 times the IQR; outliers are shown as individual points. Mean PBVC by study group (C), for patients with PBVC data at both 24 and 96 weeks (n=374); whiskers are SD. PBVC=percentage brain volume change.

Figure 2 shows the primary outcome of PBVC at 96 weeks. The adjusted mean PBVC did not differ between each active treatment and placebo (table 2). Findings of sensitivity analyses accorded with the primary analysis results (appendix p 4).

**Table 2:** Primary outcome analysis of PBVC at 96 weeks, intention-to-treat population

	**Normalised brain volume at baseline (mL)**	PBVC at 96 weeks*	**Adjusted mean difference in PBVC *vs* placebo (95% CI)**†	**p value**†
Amiloride (n=99)	1426·9 (82·4)	−1·3% (1·0)	0·0% (−0·4 to 0·5)	0·99
Fluoxetine (n=96)	1414·9 (79·9)	−1·4% (1·5)	−0·1% (−0·5 to 0·3)	0·86
Riluzole (n=99)	1416·0 (75·0)	−1·4% (1·5)	−0·1% (−0·6 to 0·3)	0·77
Placebo (n=99)	1428·8 (91·5)	−1·3% (1·1)	..	..

Data are mean (SD) unless otherwise stated. PBVC=percentage brain volume change.

* Negative values indicate reductions in brain volume.

† Adjusted for minimisation variables (age, sex, study centre, and EDSS score at randomisation) and baseline normalised brain volume, and accounted for multiple testing versus a common control group using Dunnett's method.

At 96 weeks, the mean number of new or enlarging T2 lesions in patients assigned placebo was 3·0 (SD 6·9; median 0 [IQR 0–2]). Compared with placebo, a similar number of new or enlarging T2 lesions were detected at the 96-week MRI scan for amiloride (rate ratio [RR] 1·2, 95% CI 0·8–1·8; p=0·29) and for riluzole (1·0, 0·6–1·5; p=0·81). Fluoxetine had fewer new or enlarging T2 lesions compared with placebo (RR 0·5, 95% CI 0·3–0·9; p=0·012).

The PBVC at 24 weeks was greater for patients assigned fluoxetine than for those allocated placebo (adjusted mean difference −0·31, 95% simultaneous CI −0·60 to −0·02; Dunnet-adjusted p=0·032), but not for the other active treatment groups versus placebo. No difference was noted between active treatment groups and placebo for PBVC between 24 weeks and 96 weeks (appendix p 4). Secondary outcomes accord with insufficient evidence of therapeutic effect (table 3). Although five of 60 clinician-reported and patient-reported outcome comparisons against placebo were significant (p<0·05), this finding is similar to what we would expect due to random chance when testing all 60 comparisons each at the 5% significance level.

**Table 3:** Secondary outcomes at 96 weeks

		**Amiloride (n=102)**	**Fluoxetine (n=97)**	**Riluzole (n=96)**	**Placebo (n=98)**	**Amiloride *vs* placebo**		**Fluoxetine *vs* placebo**		**Riluzole *vs* placebo**	
						Adjusted mean difference (95% CI)*	p value	Adjusted mean difference (95% CI)*	p value	Adjusted mean difference (95% CI)*	p value
Expanded Disability Status Scale score†		6·0 (1·0)	5·9 (1·2)	6·0 (1·1)	6·0 (1·1)	0·1 (−0·1 to 0·2)	0·61	−0·1 (−0·3 to 0·2)	0·54	0·1 (−0·2 to 0·2)	0·63
Multiple Sclerosis Functional Composite score (Z-score)‡		−0·55 (1·87)	−0·53 (1·66)	−0·47 (1·69)	−0·41 (1·78)	0·06 (−0·15 to 0·27)	0·59	−0·09 (−0·30 to 0·12)	0·42	0·02 (−0·19 to 0·23)	0·84
Paced Auditory Serial Addition Test		41·1 (14·3)	36·8 (16·3)	38·4 (15·6)	41·9 (16·7)	0·9 (−1·9 to 3·7)	0·51	−1·1 (−3·9 to 1·8)	0·47	0·4 (−2·4 to 3·3)	0·76
9-Hole Peg Test (s^−1^)		0·03 (0·01)	0·03 (0·01)	0·03 (0·01)	0·03 (0·01)	0·00 (−0·00 to 0·00)	0·36	0·00 (−0·00 to 0·00)	0·85	0·00 (−0·00 to 0·00)	0·27
Symbol Digit Modalities Test		43·0 (14·9)	44·8 (13·7)	44·9 (14·2)	46·1 (14·5)	−1·0 (−3·1 to 1·0)	0·32	−1·1 (−3·2 to 0·9)	0·29	−0·8 (−2·9 to 1·3)	0·44
High contrast (100%) visual acuity		50·1 (10·9)	50·5 (11·2)	47·8 (15·8)	49·1 (13·7)	3·0 (0·5 to 5·6)	0·020	1·8 (−0·7 to 4·4)	0·16	1·6 (−1·0 to 4·2)	0·22
Sloan low contrast visual acuity											
	5% contrast	31·8 (12·9)	32·6 (13·1)	31·2 (15·6)	32·4 (13·5)	1·0 (−1·8 to 3·8)	0·49	1·3 (−1·6 to 4·1)	0·38	1·5 (−1·4 to 4·3)	0·32
	2·5% contrast	17·1 (14·2)	16·5 (13·2)	17·7 (14·2)	18·1 (13·4)	0·9 (−2·0 to 3·7)	0·55	1·3 (−1·5 to 4·1)	0·37	1·7 (−1·2 to 4·6)	0·25
	1·25% contrast	5·2 (8·8)	4·0 (8·2)	6·1 (10·4)	6·8 (9·7)	−0·6 (−2·7 to 1·5)	0·58	−0·8 (−2·9 to 1·3)	0·46	0·7 (−1·4 to 2·9)	0·49
Multiple Sclerosis Impact Scale 29 items version 2											
	Total score	72·3 (16·3)	69·7 (15·1)	72·9 (15·8)	69·5 (17·2)	3·7 (0·2 to 7·2)	0·037	0·5 (−3·0 to 4·0)	0·79	0·9 (−2·6 to 4·5)	0·60
	Physical score	53·2 (12·0)	52·1 (11·4)	53·6 (12·4)	51·3 (12·7)	2·2 (−0·3 to 4·7)	0·089	0·7 (−1·8 to 3·3)	0·57	0·6 (−2·0 to 3·1)	0·66
	Psychological score	19·1 (6·1)	17·7 (5·3)	19·3 (5·4)	18·3 (5·8)	1·5 (0·2 to 2·8)	0·025	−0·3 (−1·6 to 1·0)	0·68	0·5 (−0·9 to 1·8)	0·49
Multiple Sclerosis Walking Scale version 2		44·2 (9·4)	44·4 (8·8)	44·6 (9·6)	43·6 (10·1)	0·5 (−1·6 to 2·5)	0·66	1·0 (−1·1 to 3·1)	0·35	0·6 (−1·5 to 2·8)	0·55
Neurological Fatigue Index											
	Summary score	19·4 (5·3)	18·3 (4·0)	19·7 (4·8)	18·3 (5·5)	0·9 (−0·2 to 2·0)	0·11	0·5 (−0·6 to 1·7)	0·36	0·7 (−0·4 to 1·9)	0·20
	Physical score	16·3 (4·5)	15·4 (3·8)	16·4 (4·2)	15·0 (4·8)	1·2 (0·2 to 2·3)	0·019	0·7 (−0·3 to 1·8)	0·17	1·0 (−0·0 to 2·1)	0·061
	Cognitive score	7·1 (2·5)	6·7 (2·1)	7·4 (2·1)	6·8 (2·9)	0·1 (−0·4 to 0·6)	0·70	0·2 (−0·4 to 0·7)	0·55	0·2 (−0·4 to 0·7)	0·55
	Diurnal score	10·7 (3·2)	10·3 (2·9)	10·7 (2·5)	10·6 (2·6)	−0·3 (−0·9 to 0·3)	0·36	−0·3 (−1·0 to 0·3)	0·29	−0·3 (−0·9 to 0·4)	0·40
	Nocturnal score	8·7 (2·1)	8·3 (2·3)	8·4 (2·5)	8·2 (2·2)	0·6 (0·1 to 1·1)	0·013	0·4 (−0·1 to 0·9)	0·12	0·1 (−0·4 to 0·6)	0·65
EQ-5D-5L											
	Index score	0·60 (0·23)	0·62 (0·21)	0·60 (0·19)	0·61 (0·22)	−0·01 (−0·06 to 0·04)	0·69	−0·02 (−0·06 to 0·03)	0·52	−0·01 (−0·05 to 0·04)	0·82
	VAS score	66·1 (16·9)	67·5 (19·5)	61·7 (21·0)	65·2 (20·3)	−2·1 (−7·6 to 3·5)	0·47	−3·0 (−8·6 to 2·6)	0·29	−3·0 (−8·6 to 2·7)	0·30
New or enlarging T2 lesions		3·7 (8·1)	1·8 (5·3)	2·8 (5·7)	3·0 (6·9)	1·2 (0·8 to 1·8)	0·29	0·5 (0·3 to 0·9)	0·012	1·0 (0·6 to 1·5)	0·81

Data are mean (SD). Results are derived from a model analysing data for 393 participants who had at least some 96-week outcome data. Numbers of patients are the maximum per group; the minimum sample size was n=380 for each secondary outcome (minimum per group: amiloride, n=98; fluoxetine, n=94; riluzole, n=92; placebo, n=94). The multiple regression model for each outcome included randomised treatment as an explanatory factor variable (with placebo as the reference category), the baseline measurement, and minimisation variables (age, sex, treatment centre, and Expanded Disability Status Scale score at baseline). EQ-5D-5L=EuroQol five dimensions five levels. VAS=visual analogue scale.

* Effect sizes are adjusted mean difference (95% CI), except for new or enlarging T2 lesions, which is the adjusted rate ratio (investigational drug:placebo) and 95% CI.

† 95% CIs calculated using 1000 bootstrap resamples.

‡ The Multiple Sclerosis Functional Composite score was signed square-root transformed before analysis.

51 (11%) of 445 patients had at least one relapse during the study, with 16 (14%) having a relapse in the amiloride group, ten (9%) in the fluoxetine group, 11 (10%) in the riluzole group, and 14 (12%) in the placebo group. Compared with placebo, time to first relapse did not differ for amiloride (hazard ratio [HR] 1·14, 95% CI 0·56–2·35), fluoxetine (0·74, 0·33–1·66), or riluzole (0·78, 0·35–1·73). Similarly, compared with placebo, the Timed 25-Foot Walk test did not differ for amiloride (HR 0·82, 95% CI 0·61–1·12), fluoxetine (0·81, 0·59–1·10), or riluzole (0·84, 0·61–1·13).

Findings of the clinician and patient masking questionnaire indicated that treatment assignments had been masked successfully: 51% of patients and 59% of clinicians who made a guess regarding active treatment or placebo status were correct (κ=0·04, 95% CI −0·06 to 0·14; and κ=0·13, −0·01 to 0·26; respectively). Secondary outcomes at 48 weeks are reported in the appendix (pp 11–12). Concomitant drugs taken throughout the duration of the trial are listed in the appendix (p 13).

**Figure 3:**
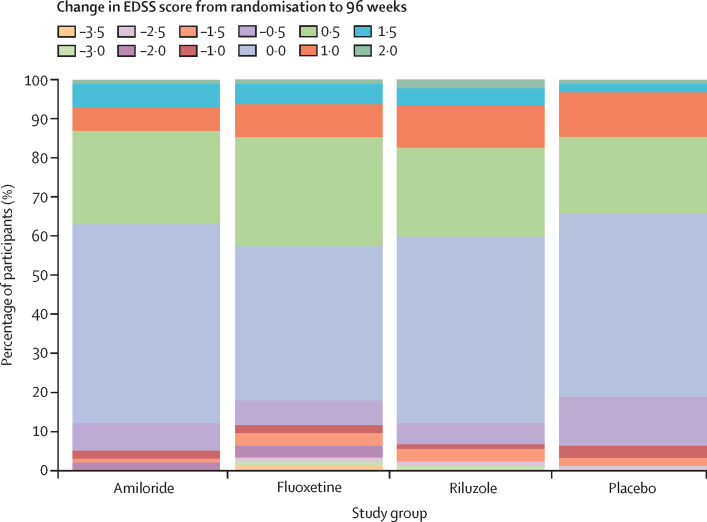
Stacked bar chart for change in EDSS score from randomisation to 96 weeks Positive change is worsening in EDSS score and negative change is improvement in EDSS score. EDSS=Expanded Disability Status Scale.

Our study population showed deterioration in several outcomes over the 96-week period (appendix pp 5–10). For example, in the placebo group, little change was noted between baseline and week 96 in the Paced Auditory Serial Addition Test, the 9-Hole Peg Test, the Symbol Digit Modalities Test, high contrast (100%) visual acuity, and Sloan low contrast visual acuity (contrast 5%), but substantial changes were seen in EDSS scores (figure 3), the Timed 25-Foot Walk, and Sloan low-contrast visual acuity (contrast 1·25%).

No emergent safety issues were recorded with the active treatments. Adverse and serious adverse events are shown in table 4 and in the appendix (pp 14, 15). Three deaths occurred during the study, which were judged by the Data Monitoring Committee unrelated to allocated treatments. One patient assigned amiloride died from metastatic lung cancer, one patient assigned riluzole died from ischaemic heart disease and coronary artery thrombosis, and one patient assigned fluoxetine had a sudden death (primary cause) with secondary causes of death listed as multiple sclerosis and obesity in the coroner's report.

**Table 4:** Adverse events, safety population

		**Amiloride (n=111)**	**Fluoxetine (n=111)**	**Riluzole (n=109)**	**Placebo (n=112)**
Adverse events (n)		609	738	634	582
Patients experiencing at least one adverse event		100 (90%)	105 (95%)	101 (93%)	103 (92%)
	Cardiac disorders	1 (1%)	3 (3%)	8 (7%)	2 (2%)
	Eye disorders	13 (12%)	8 (7%)	9 (8%)	8 (7%)
	Gastrointestinal disorders	46 (41%)	62 (56%)	49 (45%)	36 (32%)
	General disorders and administration	26 (23%)	28 (25%)	27 (25%)	32 (29%)
	Infections and infestations	68 (61%)	58 (52%)	62 (57%)	69 (62%)
	Injury, poisoning, and procedural complications	26 (23%)	43 (39%)	29 (27%)	28 (25%)
	Investigations*	10 (9%)	20 (18%)	17 (16%)	8 (7%)
	Metabolism and nutrition disorders	2 (2%)	9 (8%)	7 (6%)	4 (4%)
	Musculoskeletal and connective tissue disorders	37 (33%)	26 (23%)	37 (34%)	29 (26%)
	Nervous system disorders	48 (43%)	46 (41%)	47 (43%)	44 (39%)
	Psychiatric disorders	21 (19%)	30 (27%)	22 (20%)	22 (20%)
	Renal and urinary disorders	9 (8%)	13 (12%)	10 (9%)	5 (4%)
	Respiratory disorders	15 (14%)	23 (21%)	13 (12%)	16 (14%)
	Skin and subcutaneous tissue disorders	16 (14%)	11 (10%)	13 (12%)	17 (15%)
	Surgical and medical procedures	6 (5%)	3 (3%)	8 (7%)	7 (6%)
	Vascular disorders	4 (4%)	2 (2%)	3 (3%)	6 (5%)
Patients experiencing at least one serious adverse event		10 (9%)	7 (6%)	12 (11%)	13 (12%)
	Infections and infestations	4 (4%)	1 (1%)	4 (4%)	4 (4%)
	Injury, poisoning, and procedural complications	3 (3%)	0 (0%)	3 (3%)	2 (2%)
Patients experiencing at least one suspected unexplained serious adverse reaction		0 (0%)	0 (0%)	1 (1%)	0 (0%)

Data are number of patients experiencing each type of event (% of cohort). Adverse events occurring in at least 5% of patients in any study group are shown. Serious adverse events occurring in at least 3% of patients in any group are shown. Full data are provided in the appendix (pp 14–15). The safety population comprised all patients who underwent randomisation, excluding two patients allocated riluzole who were prescribed fluoxetine by their family doctor towards the end of the trial (protocol deviation); these patients had five adverse events and no serious adverse events; a few of the adverse events occurred after fluoxetine was prescribed and, therefore, might be attributable to fluoxetine rather than riluzole (or a combination of the two). Progressive change due to secondary progressive multiple sclerosis in motor, sensory, balance, sphincter (including urinary tract infections), vision, cognitive, and fatigue levels were not reported as adverse events, serious adverse events, or suspected unexplained serious adverse reactions. Relapses were not reported as adverse events, serious adverse events, or suspected unexplained serious adverse reactions but are collated separately.

* For example, abnormal blood results or weight loss.

## Discussion

The findings of MS-SMART, a large, multicentre, multiarm randomised trial in patients with secondary progressive multiple sclerosis, showed that none of the three study drugs (amiloride, fluoxetine, and riluzole) had any effect on the primary outcome of PBVC over 96 weeks or any secondary outcomes, compared with placebo. Significant efficiency gains with respect to the study design were, however, seen in terms of people, time, and economic resources. The study cohort was typical of patients with non-relapsing secondary progressive multiple sclerosis, with a median disease duration of 21 years (IQR 15–29), median secondary progression of 6 years (3–10), use of unilateral support when walking (median EDSS score 6·0 [5·5–6·5]), and more than 90% of patients were relapse-free for at least 2 years before recruitment. High levels of retention (>90%) and adherence were reported, and on-trial behaviour was also typical of patients with non-relapsing secondary progressive multiple sclerosis, with low levels of inflammatory disease activity clinically (11% of patients had an on-study relapse) and radiologically (mean number of new or enlarging lesions was 3·0 [SD 6·9], median 0 [IQR 0–2]). Clear radiological progression was seen, with PBVC of roughly −0·7% per year.

Taken together with the effectiveness of our trial masking and the meeting of all previous assumptions underlying our power calculations, MS-SMART was sensitive to detect any neuroprotective effect of the three drugs tested. Some data were missing, 12% for the primary endpoint and as high as 15% for some secondary endpoints; although this shortfall brings some potential for bias, sensitivity analyses were supportive of the findings of the primary analysis.

Drug selection in our study was based on findings of two separate systematic reviews:^6^ first, we looked at preclinical studies of demyelination, inflammation, axonal loss, and neurobehavioural changes in EAE models of multiple sclerosis; second, we assessed clinical evidence at phase 2a in patients with multiple sclerosis and other neurodegenerative disorders that share common pathways of neurodegeneration. We did not select agents with clinically significant safety profile issues, those most likely to produce only symptomatic benefit, drugs with an immunosuppressive mechanism of action, agents with limited efficacy data or biological plausibility, and those that had been assessed previously in patients with relapsing-remitting multiple sclerosis.

After initial identification of 120 potential candidates, two further important review steps led us to identify seven drugs for analysis: ibudilast, oxcarbazepine, pirfenidone, polyunsaturated fatty acids (including lipoic acid), amiloride, fluoxetine, and riluzole. Ibudilast and lipoic acid have now shown phase 2 success, confirming the predictive value of our candidate selection methodology.^7^, ^8^ Pharmacokinetic considerations (eg, limited bioavailability in the CNS) are also of potential relevance to the results of our trial. However, riluzole and fluoxetine have known CNS effects at the doses used and are established drugs for treatment of motor neuron disease and depression, respectively. Amiloride has a primary non-CNS target; however, the dose used was based on an earlier phase 2a trial.

The mechanisms of action of the three drugs we tested (accepting that there are likely to be additional off-target effects of these drugs) mainly target distinct possible axonal pathobiological features implicated in the neurodegenerative substrate of progressive multiple sclerosis. Amiloride blocks acid-sensing ion channels and, therefore, targets axonal calcium overload; riluzole targets glutamate-mediated excitotoxic injury; and fluoxetine stimulates astrocytic lactate release. Fluoxetine aims to provide essential energy substrates to neurons.^13^, ^14^

Although all these processes represent biologically plausible targets for neuroprotection, many additional pathological processes can be implicated in disease progression, including innate or adaptive mediated inflammation and inadequate remyelination. Thus, several explanations could account for why amiloride, fluoxetine, and riluzole did not show efficacy in our study, including (but not restricted to) the ultimate relevance of the dynamic in-vivo disease processes primarily targeted by these three drugs. Indeed, our findings expose our incomplete knowledge of the pathobiology of secondary progressive multiple sclerosis.

Moreover, although evidence (clinical and MRI) for ongoing focal inflammatory disease activity in our study cohort was scant, we did not record any B-cell or microglial activity. In terms of phase 2 neuroprotection trials showing reductions in the rate of brain atrophy of 40–70%, use of disease-modifying therapies has been between 0% and 45%.^7^, ^8^, ^30^ In view of positive results from the ocrelizumab^4^ and siponimod^3^ phase 3 trials, future work will most likely stratify use of anti-inflammatory compounds in conjunction with possible neuroprotective agents.

The findings that fluoxetine was associated not only with a significantly higher PBVC at 24 weeks compared with placebo but also a concomitant decrease in the number of new or enlarging T2 lesions at 96 weeks should be interpreted with caution. These outcomes were secondary outcomes and the number of participants developing new or enlarging T2 lesion was, overall, low (mean 3·0 [SD 6·9] lesions; median 0 [IQR 0–2]).

Additionally, the MRI protocol did not include a post-gadolinium scan at baseline and we were, therefore, unable to establish whether some of the patients allocated fluoxetine had, by chance, greater ongoing subclinical disease inflammatory activity at study entry. A paradoxical reduction in parenchymal brain volume compared with placebo (pseudoatrophy) has been well described^31^, ^35^, ^36^ in both immunomodulatory (eg, natalizumab)^35^ and sodium-channel blockade (eg, lamotrigine)^31^ studies. Fluoxetine might, therefore, exert a degree of immunomodulatory activity (eg, decreased lymphocyte proliferation and suppressed interferon-γ production) by acting on astrocytes;^37^ findings of a small study showed reduction of new enhancing lesions in patients with relapsing-remitting multiple sclerosis.^38^ However, the primary outcome of our study at week 96 was negative and accords with the lack of therapeutic effect seen in the FLUOX-PMS trial of 137 patients with progressive multiple sclerosis, which also did not show a beneficial effect on brain atrophy in a subgroup.^39^

The MS-SMART trial was restricted to patients with secondary progressive multiple sclerosis, with inclusion criteria as described. Generalisation to a wider population (eg, greater disability, older, or with primary progressive disease) is uncertain. Nonetheless, overall, we feel our study population will have mostly captured the secondary progressive clinical phenotype.

In conclusion, we have shown that a multiarm approach can be used successfully to expedite drug discovery in patients with progressive multiple sclerosis. Such trial designs will be highly relevant to future therapeutic development in brain medicine in general. They are necessary to confirm or refute postulated pathways and give insight into the pathobiology of progressive multiple sclerosis.

Our results do not support the effectiveness of amiloride, fluoxetine, and riluzole in reducing disease progression for secondary progressive multiple sclerosis, and they indicate that exclusive targeting of axonal pathobiology is an inadequate strategy to achieve neuroprotection in progressive disease. In view of the need to develop disease-modifying treatments for progressive multiple sclerosis, this finding challenges the area of research to shift towards combinatorial strategies or stratification based on greater resolution of relevant pathobiology at the level of individual patients.

## Data sharing

The MS-SMART study protocol and statistical analysis plan are available on request to the chief investigator (Prof Jeremy Chataway; j.chataway@ucl.ac.uk). All data requests should be submitted to JC for consideration in the first instance. Access to available fully anonymised data may be granted 12 months after publication, after review by JC and the sponsor (University College London). Requesters will be asked to complete an application form detailing specific requirements, rationale, and proposed use. A data-sharing agreement will need to be signed. Requested data will be made available, along with supporting documentation (eg, data dictionary) on a secure server.
