# Supplementary material for: Efficacy of three neuroprotective drugs in secondary progressive multiple sclerosis (MS-SMART): a phase 2b, multiarm, double-blind, randomised placebo-controlled trial
Source: Lancet Neurol. 2020 Mar;19(3):214–25. doi: 10.1016/S1474-4422(19)30485-5 (PMC7029307; doi:10.1016/S1474-4422(19)30485-5)
Supplement: Supplementary appendix [file mmc1.pdf]

THE LANCET
Neurology

Supplementary appendix

This appendix formed part of the original submission and has been peer reviewed. We post it as supplied by the authors.

Supplement to: Ontaneda D, Tallantyre E, Kalincik T, Planchon SM, Evangelou N. Early highly effective versus escalation treatment approaches in relapsing multiple sclerosis. *Lancet Neurol* 2019; published online July 30. http://dx.doi.org/10.1016/S1474-4422(19)30151-6.

SUPPLEMENTARY APPENDIX

This appendix has been provided by the authors to give additional information about their study.

Supplement to: Chataway J, De Angelis F, Connick P, et al. *Multiple Sclerosis-Secondary Progressive Multi-Arm Randomisation Trial (MS-SMART): a multi-arm phase 2B randomised, double-blind, placebo-controlled clinical trial comparing the efficacy of three neuroprotective drugs in secondary progressive multiple sclerosis.*

Contents

Inclusion Criteria......2

Exclusion Criteria......3

Table S1: Sensitivity analyses and secondary analyses of Percentage Brain Volume Change.4

Table S2: Summary statistics for the clinical secondary outcomes, split by trial arm......5

Table S3: Secondary outcomes at 48 weeks, split by trial arm......11

Table S4: Concomitant medications......13

Table S5: Full table of adverse events occurring in the safety population......14

Inclusion Criteria

- Confirmed diagnosis of secondary progressive multiple sclerosis (SPMS). Steady progression rather than relapse must be the major cause of increasing disability in the preceding 2 years. Progression can be evident from either an increase of at least one point in the expanded disability status scale (EDSS) or clinical documentation of increasing disability in patients notes
- EDSS 4.0-6.5
- Aged 25 to 65 inclusive
- Women and men with partners of childbearing potential must be using an appropriate method of contraception to avoid any unlikely teratogenic effects of the 3 drugs from time of consent, to 6 weeks after treatment inclusive
- Women must have a negative pregnancy test within 7 days prior to the baseline visit unless not of child bearing potential (e.g. have undergone a hysterectomy, bilateral tubal ligation or bilateral oophorectomy or they are postmenopausal)
- Willing and able to comply with the trial protocol (e.g. can tolerate magnetic resonance imaging [MRI] and fulfils the requirements for MRI, e.g. not fitted with pacemakers or permanent hearing aids) ability to understand and complete questionnaires
- Written informed consent

Exclusion Criteria

- Pregnancy or breastfeeding females
- Baseline MRI scan not of adequate quality for analysis (e.g. too much movement artefact)
- Significant organ co-morbidity (e.g. malignancy or renal or hepatic failure)
- Relapse within 3 months of baseline visit
- Patients who have been treated with iv or oral steroids for an multiple sclerosis relapse/progression within 3 months of baseline visit (these patients can undergo future screening visits once the 3 month window has expired), patients on steroids for another medical condition may enter as long as the steroid prescription is not for multiple sclerosis (relapse/ progression).
- Use of Simvastatin at 80 mg dose within 3 months of baseline visit (lower doses of Simvastatin and other statins are permissible)
- Commencement of fampridine within 6 months of baseline visit

Table S1: Sensitivity analyses and secondary analyses of Percentage Brain Volume Change

		Amiloride-Placebo			Fluoxetine-Placebo			Riluzole- placebo		
**Analysis**	**N**	**Adjusted Mean difference**	**Simultaneous 95% Confidence Interval**	**Dunnett adjusted p-value**	**Adjusted Mean difference**	**Simultaneous 95% Confidence Interval**	**Dunnett adjusted p-value**	**Adjusted Mean difference**	**Simultaneous 95% Confidence Interval**	**Dunnett adjusted p-value**
Sensitivity analysis 1: Multiple Imputation based on a MNAR assumption*	445	0.064	-0.376 to 0.503	0.776	-0.137	-0.575 to 0.302	0.541	-0.120	-0.562 to 0.322	0.594
Sensitivity analysis 2: Multiple Imputation based on a MAR assumption	445	0.039	-0.386 to 0.464	0.858	-0.108	-0.528 to 0.311	0.613	-0.129	-0.550 to 0.292	0.549
Sensitivity analysis 3: with outliers removed	392	0.041	-0.374 to 0.456	0.991	-0.052	-0.472 to 0.368	0.983	-0.139	-0.555 to 0.277	0.770
Secondary analysis: using per protocol population	263	0.122	-0.360 to 0.605	0.883	-0.050	-0.539 to 0.439	0.991	0.097	-0.395 to 0.588	0.940
Secondary analysis: PBVC at 24 weeks	404	-0.006	-0.297 to 0.285	1.000	-0.308	-0.596 to -0.020	**0.032**	-0.179	-0.473 to 0.115	0.342
Secondary analysis: PBVC between 24 and 96 weeks	360	0.083	-0.336 to 0.501	0.938	0.221	-0.199 to 0.641	0.461	0.150	-0.269 to 0.569	0.733

The multiple regression model for each outcome included trial arm as an explanatory factor variable (with placebo as the reference category), baseline normalised brain volume, and the minimisation variables: age, gender, treatment centre and EDSS at randomisation. This was the same model as used for the primary analysis of the primary outcome. EDSS: expanded disability status scale, MAR: Missing-At-Random, MNAR: Missing-Not-At-Random, PBVC: Percentage Brain Volume Change.

Table S2: Summary statistics for the clinical secondary outcomes, split by trial arm

			Allocated Treatment				
			**Amiloride**	**Fluoxetine**	**Riluzole**	**Placebo**	**Overall**
EDSS, score							
	Baseline	N	111	111	111	112	445
		Mean	5.878	5.901	5.838	5.871	5.872
		SD	0.745	0.710	0.748	0.773	0.742
	48 weeks	N	103	98	97	100	398
		Mean	6.005	5.985	6.036	5.905	5.982
		SD	0.769	0.889	0.814	0.909	0.845
	96 weeks	N	100	95	92	96	383
		Mean	6.030	5.932	5.962	5.953	5.970
		SD	1.002	1.189	1.072	1.021	1.069
MSFC, z-score							
	Baseline	N	111	111	111	112	445
		Mean	-0.191	-0.016	-0.092	-0.003	-0.075
		SD	1.189	0.602	0.952	0.906	0.935
	48 weeks	N	103	97	96	99	395
		Mean	-0.211	-0.249	-0.328	-0.213	-0.249
		SD	1.255	1.089	1.477	1.357	1.297
	96 weeks	N	100	95	95	97	387
		Mean	-0.548	-0.528	-0.473	-0.409	-0.490
		SD	1.868	1.664	1.687	1.776	1.746
PASAT, no. of correct answers							
	Baseline	N	111	110	111	112	444
		Mean	39.018	36.600	36.910	41.464	38.509
		SD	13.667	15.171	16.003	13.864	14.784
	48 weeks	N	103	97	94	98	392
		Mean	38.049	36.361	39.936	41.459	38.936
		SD	15.428	15.710	14.250	14.092	14.965
	96 weeks	N	99	95	94	97	385
		Mean	41.061	36.779	38.394	41.907	39.566
		SD	14.340	16.322	15.554	16.728	15.826
T25FW, s							
	Baseline	N	111	109	111	112	443
		Mean	25.136	15.699	19.836	18.633	19.842

			Allocated Treatment				
			Amiloride	Fluoxetine	Riluzole	Placebo	Overall
		SD	38.843	12.381	28.459	28.530	28.819
	48 weeks	N	100	96	96	99	391
		Mean	25.510	23.639	31.248	26.498	26.710
		SD	38.401	31.874	49.335	43.941	41.323
	96 weeks	N	99	94	93	96	382
		Mean	38.988	33.899	33.953	32.882	34.976
		SD	56.050	50.680	52.529	52.372	52.831
9HPT, s^{-1}							
	Baseline	N	111	111	111	112	445
		Mean	0.034	0.034	0.034	0.033	0.034
		SD	0.011	0.010	0.010	0.010	0.010
	48 weeks	N	103	97	96	99	395
		Mean	0.034	0.032	0.034	0.033	0.034
		SD	0.012	0.010	0.010	0.011	0.011
	96 weeks	N	100	95	95	97	387
		Mean	0.033	0.033	0.034	0.033	0.033
		SD	0.012	0.011	0.012	0.012	0.012
SDMT, no. of correct answers							
	Baseline	N	109	111	109	112	441
		Mean	43.908	44.153	44.514	44.107	44.170
		SD	12.400	11.410	13.065	12.778	12.386
	48 weeks	N	101	97	95	99	392
		Mean	43.584	44.454	45.105	44.960	44.515
		SD	14.505	12.177	13.075	13.093	13.215
	96 weeks	N	100	95	93	94	382
		Mean	42.960	44.779	44.892	46.096	44.654
		SD	14.906	13.743	14.219	14.480	14.339
HCVA (100%), no. of correct answers							
	Baseline	N	107	111	110	111	439
		Mean	50.075	50.838	48.473	50.378	49.943
		SD	11.193	10.801	14.836	12.668	12.470
	48 weeks	N	99	96	95	98	388
		Mean	51.000	50.688	49.642	50.520	50.469
		SD	9.876	10.805	14.730	11.875	11.902
	96 weeks	N	98	96	93	95	382
		Mean	50.082	50.490	47.806	49.095	49.385

			Allocated Treatment				
			Amiloride	Fluoxetine	Riluzole	Placebo	Overall
		SD	10.914	11.161	15.812	13.696	13.004
SLCVA (5%), no. of correct answers							
	Baseline	N	107	110	108	110	435
		Mean	32.579	32.927	29.963	33.927	32.359
		SD	13.401	12.820	16.131	14.575	14.311
	48 weeks	N	98	96	95	97	386
		Mean	31.480	30.427	32.747	32.258	31.725
		SD	13.699	12.096	14.824	14.264	13.731
	96 weeks	N	98	95	93	94	380
		Mean	31.827	32.611	31.151	32.415	32.003
		SD	12.906	13.072	15.650	13.470	13.759
SLCVA (2.5%), no. of correct answers							
	Baseline	N	107	111	109	111	438
		Mean	19.103	17.649	18.807	20.847	19.103
		SD	12.709	12.356	14.164	13.978	13.331
	48 weeks	N	98	96	95	98	387
		Mean	18.204	14.458	20.168	18.755	17.897
		SD	13.376	12.115	14.435	13.656	13.532
	96 weeks	N	98	96	93	95	382
		Mean	17.133	16.521	17.656	18.084	17.343
		SD	14.209	13.160	14.228	13.378	13.710
SLCVA (1.25%), no. of correct answers							
	Baseline	N	107	111	109	111	438
		Mean	8.168	6.937	7.083	9.865	8.016
		SD	10.670	9.592	10.657	11.878	10.759
	48 weeks	N	98	96	94	98	386
		Mean	7.000	4.833	7.660	8.398	6.977
		SD	10.045	8.347	11.074	10.845	10.181
	96 weeks	N	98	96	93	95	382
		Mean	5.184	4.042	6.065	6.779	5.508
		SD	8.836	8.173	10.351	9.725	9.316
MSISv2 Total, score							
	Baseline	N	111	111	111	112	445
		Mean	63.915	65.000	69.154	66.072	66.036
		SD	13.374	13.828	15.011	14.389	14.251
		N	104	95	98	102	399

			Allocated Treatment				
			Amiloride	Fluoxetine	Riluzole	Placebo	Overall
	48 weeks	Mean	68.630	66.232	71.677	69.357	68.993
		SD	15.935	14.817	14.873	15.749	15.430
	96 weeks	N	101	96	96	97	390
		Mean	72.332	69.749	72.902	69.533	71.140
		SD	16.335	15.117	15.799	17.173	16.137
MSISv2 Psychological, score							
	Baseline	N	111	111	111	112	445
		Mean	15.947	16.657	18.163	17.103	16.968
		SD	4.511	4.759	5.427	4.977	4.979
	48 weeks	N	104	95	98	102	399
		Mean	17.721	16.737	19.158	18.444	18.024
		SD	5.620	5.213	5.255	5.352	5.420
	96 weeks	N	101	96	96	97	390
		Mean	19.108	17.676	19.333	18.256	18.599
		SD	6.063	5.257	5.352	5.799	5.651
MSISv2 Physical, score							
	Baseline	N	111	111	111	112	445
		Mean	47.968	48.344	50.991	48.969	49.068
		SD	10.498	10.439	11.300	11.224	10.899
	48 weeks	N	104	96	98	102	400
		Mean	50.909	49.517	52.519	50.914	50.971
		SD	12.060	10.908	10.961	11.785	11.460
	96 weeks	N	101	97	96	98	392
		Mean	53.225	52.052	53.569	51.273	52.531
		SD	12.023	11.399	12.411	12.700	12.131
MSWSv2 Total, score							
	Baseline	N	111	111	110	112	444
		Mean	41.428	41.078	42.641	41.633	41.693
		SD	9.182	9.773	9.319	9.935	9.544
	48 weeks	N	104	95	98	102	399
		Mean	42.664	42.263	44.738	43.372	43.259
		SD	10.122	9.352	8.053	9.919	9.424
	96 weeks	N	102	96	96	96	390
		Mean	44.188	44.363	44.583	43.646	44.195
		SD	9.445	8.804	9.620	10.134	9.481
NFI Summary Interval, score			111	110	109	108	438

			Allocated Treatment				
			Amiloride	Fluoxetine	Riluzole	Placebo	Overall
	Baseline	N					
		Mean	18.013	17.430	19.052	17.803	18.074
		SD	4.198	3.890	4.799	3.856	4.232
	48 weeks	N	104	93	98	101	396
		Mean	18.474	17.874	19.420	18.202	18.498
		SD	5.250	3.693	4.453	4.246	4.486
	96 weeks	N	100	97	96	98	391
		Mean	19.384	18.326	19.708	18.337	18.938
		SD	5.275	3.988	4.828	5.451	4.942
NFI Physical Interval, score							
	Baseline	N	111	111	109	110	441
		Mean	15.116	14.663	15.907	14.740	15.104
		SD	3.780	3.869	4.339	3.654	3.935
	48 weeks	N	104	94	98	101	397
		Mean	15.340	14.939	16.238	15.307	15.458
		SD	4.582	3.365	3.799	3.984	3.986
	96 weeks	N	101	97	96	98	392
		Mean	16.323	15.424	16.381	14.991	15.782
		SD	4.535	3.801	4.213	4.842	4.392
NFI Cognitive Interval, score							
	Baseline	N	111	110	109	109	439
		Mean	6.517	6.215	7.065	6.306	6.525
		SD	2.207	2.243	2.331	2.380	2.307
	48 weeks	N	104	93	98	101	396
		Mean	6.905	6.583	7.076	6.609	6.796
		SD	2.523	2.070	2.163	2.334	2.286
	96 weeks	N	101	97	96	98	392
		Mean	7.094	6.768	7.410	6.757	7.007
		SD	2.544	2.059	2.092	2.889	2.429
NFI Diurnal Interval, score							
	Baseline	N	111	111	110	110	442
		Mean	10.068	9.611	10.244	9.679	9.900
		SD	2.780	2.932	3.104	2.504	2.841
	48 weeks	N	102	93	97	101	393
		Mean	10.012	10.299	10.214	10.342	10.214
		SD	2.764	2.447	2.479	2.457	2.537

			Allocated Treatment				
			Amiloride	Fluoxetine	Riluzole	Placebo	Overall
	96 weeks	N	98	96	96	96	386
		Mean	10.664	10.272	10.686	10.609	10.558
		SD	3.240	2.851	2.475	2.582	2.799
NFI Nocturnal Interval, score							
	Baseline	N	111	111	111	111	444
		Mean	7.748	7.599	8.243	7.961	7.888
		SD	2.037	2.463	2.782	2.294	2.413
	48 weeks	N	104	94	98	102	398
		Mean	8.109	8.077	8.374	8.166	8.181
		SD	2.180	2.213	2.633	2.296	2.330
	96 weeks	N	100	97	96	97	390
		Mean	8.690	8.347	8.413	8.171	8.407
		SD	2.133	2.252	2.471	2.202	2.265
EQ-5D-5L index, score							
	Baseline	N	111	111	110	111	443
		Mean	0.683	0.702	0.658	0.671	0.678
		SD	0.170	0.156	0.175	0.185	0.172
	48 weeks	N	103	94	98	102	397
		Mean	0.648	0.658	0.617	0.645	0.642
		SD	0.192	0.174	0.207	0.186	0.190
	96 weeks	N	102	96	96	98	392
		Mean	0.603	0.615	0.596	0.613	0.607
		SD	0.227	0.206	0.190	0.221	0.211
EQ5D VAS: Health State, score							
	Baseline	N	111	111	111	112	445
		Mean	66.126	67.523	61.730	65.241	65.155
		SD	16.901	19.478	21.009	20.335	19.547
	48 weeks	N	103	93	97	102	395
		Mean	63.563	66.140	58.608	62.961	62.797
		SD	21.224	18.584	20.666	22.427	20.913
	96 weeks	N	102	97	96	96	391
		Mean	61.765	61.938	59.625	64.281	61.900
		SD	20.557	20.698	21.547	21.745	21.115

EDSS, Expanded Disability Status Scale; EQ-5D-5L, EuroQol-5 dimension; HCVA, high contrast visual acuity; MSFC, Multiple Sclerosis Functional Composite score; MSISv2, Multiple Sclerosis Impact Scale 29 version 2; MSWSv2, Multiple Sclerosis Walking Scale v2; NFI, Neurological Fatigue Index; SDMT, Symbol Digit Modalities Test; SLCVA, Sloan Low Contrast Visual Acuity; T25FW Timed 25 Foot Walk; 9HPT, 9 Hole Peg Test.

Table S3: Secondary outcomes at 48 weeks, split by trial arm

	**Amiloride** N=104	**Fluoxetine** N=98	**Riluzole** N=98	**Placebo** N=102	**Adjusted mean difference (Amiloride – Placebo) with 95% CI**	**P-value**	**Adjusted mean difference (Fluoxetine – Placebo) with 95% CI**	**P-value**	**Adjusted mean difference (Riluzole – Placebo) with 95% CI**	**P-value**
EDSS*, score	6.0 (0.8)	6.0 (0.9)	6.0 (0.8)	5.9 (0.9)	0.1 (0.0 to 0.3)	0.042	0.1 (-0.1 to 0.2)	0.406	0.2 (0.1 to 0.3)	0.007
MSFC**, z-score	-0.2 (1.3)	-0.2 (1.1)	-0.3 (1.5)	-0.2 (1.4)	0.06 (-0.11 to 0.24)	0.488	-0.09 (-0.27 to 0.09)	0.308	0.01 (-0.17 to 0.19)	0.913
PASAT, no. of correct answers	38.0 (15.4)	36.4 (15.7)	39.9 (14.3)	41.5 (14.1)	-0.7 (-3.1 to 1.7)	0.577	-0.6 (-3.1 to 1.8)	0.616	2.1 (-0.4 to 4.5)	0.106
9HPT, s^{-1}	0.03 (0.01)	0.03 (0.01)	0.03 (0.01)	0.03 (0.01)	0.00 (-0.001 to 0.001)	0.999	-0.001 (-0.002 to 0.001)	0.242	0.00 (-0.001 to 0.002)	0.544
SDMT, no. of correct answers	43.6 (14.5)	44.5 (12.2)	45.1 (13.1)	45.0 (13.1)	-0.6 (-2.4 to 1.3)	0.541	-1.0 (-2.9 to 0.9)	0.290	-0.3 (-2.2 to 1.6)	0.774
HCVA 100%, no. of correct answers	51.0 (9.9)	50.7 (10.8)	49.6 (14.7)	50.5 (11.9)	0.7 (-1.5 to 3.0)	0.538	-0.3 (-2.6 to 2.0)	0.799	1.1 (-1.2 to 3.3)	0.363
SLCVA 5%, no. of correct answers	31.5 (13.7)	30.4 (12.1)	32.7 (14.8)	32.3 (14.3)	-0.6 (-3.2 to 2.0)	0.656	-1.3 (-4.0 to 1.3)	0.317	2.6 (-0.1 to 5.3)	0.056
SLCVA 2·5%, no. of correct answers	18.2 (13.4)	14.5 (12.1)	20.2 (14.4)	18.8 (13.7)	0.6 (-1.9 to 3.1)	0.643	-1.8 (-4.3 to 0.7)	0.154	2.5 (0.0 to 5.1)	0.049
SLCVA 1·25%, no. of correct answers	7.0 (10.0)	4.8 (8.3)	7.7 (11.1)	8.4 (10.8)	-0.7 (-3.0 to 1.7)	0.564	-1.7 (-4.1 to -0.7)	0.159	0.2 (-2.2 to 2.6)	0.882
MSIS-29v2 Total, score	68.6 (15.9)	66.2 (14.8)	71.7 (14.9)	69.4 (15.7)	0.4 (-2.8 to 3.6)	0.795	-2.2 (-5.5 to 1.1)	0.183	0.5 (-2.7 to 3.8)	0.747
Physical, score	50.9 (12.1)	49.5 (10.9)	52.5 (11.0)	50.9 (11.8)	0.5 (-1.8 to 2.8)	0.695	-1.0 (-3.4 to 1.3)	0.398	0.5 (-1.8 to 2.9)	0.660
Psychological, score	17.7 (5.6)	16.7 (5.2)	19.2 (5.3)	18.4 (5.4)	-0.1 (-1.3 to 1.1)	0.874	-1.3 (-2.5 to -0.0)	0.045	0.1 (-1.1 to 1.3)	0.894
MSWSv2, score	42.6 (10.1)	42.3 (9.4)	44.7 (8.1)	43.4 (9.9)	-0.7 (-2.6 to 1.2)	0.456	-0.9 (-2.8 to 1.1)	0.378	1.0 (-0.9 to 2.9)	0.301
NFI Summary, score	18.5 (5.3)	17.9 (3.7)	19.4 (4.5)	18.2 (4.2)	0.2 (-0.8 to 1.2)	0.707	0.3 (-0.7 to 1.3)	0.558	0.6 (-0.4 to 1.6)	0.266
Physical, score	15.3 (4.6)	14.9 (3.4)	16.2 (3.8)	15.3 (4.0)	-0.0 (-1.0 to 0.9)	0.934	-0.1 (-1.0 to 0.9)	0.917	0.5 (-0.4 to 1.5)	0.292
Cognitive, score	6.9 (2.5)	6.6 (2.1)	7.1 (2.2)	6.6 (2.3)	0.1 (-0.3 to 0.6)	0.584	0.2 (-0.2 to 0.7)	0.333	-0.1 (-0.5 to 0.4)	0.809
Diurnal, score	10.0 (2.8)	10.3 (2.4)	10.2 (2.5)	10.3 (2.5)	-0.6 (-1.1 to -0.1)	0.032	0.0 (-0.5 to 0.6)	0.957	-0.4 (-1.0 to 0.1)	0.137
Nocturnal, score	8.1 (2.2)	8.1 (2.2)	8.4 (2.6)	8.2 (2.3)	0.1 (-0.4 to 0.6)	0.700	0.3 (-0.2 to 0.8)	0.209	0.1 (-0.4 to 0.6)	0.706
EQ-5D-5L Index, score	0.6 (0.2)	0.7 (0.2)	0.6 (0.2)	0.6 (0.2)	0.00 (-0.04 to 0.05)	0.874	0.00 (-0.04 to 0.05)	0.916	-0.02 (-0.06 to 0.03)	0.482
VAS, score	63.6 (21.2)	66.1 (18.6)	58.6 (20.7)	63.0 (22.4)	0.7 (-4.5 to 5.9)	0.796	2.6 (-2.7 to 8.0)	0.329	-2.7 (-8.0 to 2.6)	0.315

The results for each outcome were derived from a single model analysing the N=402 participants in whom at least some 48 week outcome was recorded. N's shown are maximums per group. The minimum sample size was N=386 for each secondary outcome (minimum per group: Amiloride 98, Fluoxetine 93, Riluzole 94, Placebo 97). Data presented are mean (SD). The multiple regression model for each outcome included trial arm as an explanatory factor variable (with placebo as the reference category), the baseline measurement, and the minimisation variables: age, gender, treatment centre and EDSS at randomisation. Results from the Cox proportional hazards model used to analyse T25FW at 48 weeks showed no significant differences compared with placebo (hazard ratios 0.74 (0.55 to 1.00), p=0.052; 0.81 (95% CI 0.60 to 1.09), p=0.162; 0.78 (95% CI 0.58 to 1.05), p=0.100; for amiloride, fluoxetine, riluzole respectively). 9HPT: Nine Hole Peg Test, CI: confidence interval, EDSS: Expanded Disability Status Scale, MSFC: Multiple Sclerosis Functional Composite, MSIS-29v2: Multiple Sclerosis Impact Scale 29 items version 2, MSWSv2: Multiple Sclerosis Walking Scale version 2, NFI: Neurological Fatigue Index, PASAT: Paced Auditory Serial Addition Test, SDMT: Symbol Digit Modalities Test, SLCVA: Sloan Low Contrast Visual Acuity, T25FW: timed 25-foot walk; VAS: Visual Analogue Scale.
*Confidence intervals calculated using 1000 bootstrap resamples.
**MSFC was signed-square root transformed prior to analysis.

Table S4: Concomitant medications

	Allocated Treatment									
	Amiloride		Fluoxetine		Riluzole		Placebo		Overall	
	N	%	N	%	N	%	N	%	N	%
Total number of patients randomised	111	100.0	111	100.0	111	100.0	112	100.0	445	100.0
Concomitant medications category:										
Anti-depressants	7	6.3	5	4.5	14	12.6	10	8.9	36	8.1
Anti-emetics	1	0.9	2	1.8	3	2.7	3	2.7	9	2.0
Anti-fatigue medication	10	9.0	12	10.8	9	8.1	3	2.7	34	7.6
Anti-hypertensives	15	13.5	15	13.5	18	16.2	21	18.8	69	15.5
Antibiotics	36	32.4	43	38.7	31	27.9	31	27.7	141	31.7
Asthma agents	6	5.4	5	4.5	5	4.5	2	1.8	18	4.0
Bisphosphonates	2	1.8	3	2.7	1	0.9	4	3.6	10	2.2
Bladder agents	39	35.1	49	44.1	41	36.9	48	42.9	177	39.8
Hormone Replacement Therapy (HRT)	8	7.2	7	6.3	6	5.4	12	10.7	33	7.4
Low dose naltrexone (LDN)	5	4.5	6	5.4	3	2.7	2	1.8	16	3.6
Neuropathic analgesia	44	39.6	36	32.4	47	42.3	47	42.0	174	39.1
Non-neuropathic analgesia	48	43.2	51	45.9	47	42.3	51	45.5	197	44.3
Other	76	68.5	79	71.2	92	82.9	82	73.2	329	73.9
Proton pump inhibitors (PPIs)	15	13.5	13	11.7	15	13.5	9	8.0	52	11.7
Spasticity agents	47	42.3	40	36.0	46	41.4	38	33.9	171	38.4
Statins	18	16.2	15	13.5	19	17.1	14	12.5	66	14.8
Thyroxine	6	5.4	7	6.3	14	12.6	10	8.9	37	8.3
Vitamin D supplements	52	46.8	58	52.3	51	45.9	50	44.6	211	47.4

Number of patients taking at least one concomitant medication in each concomitant medications category throughout the duration of the study (from baseline to end of follow-up), split by trial arm and overall

Table S5: Full table of adverse events occurring in the safety population

		Fluoxetine (N=111)	Riluzole (N=109)	Amiloride (N=111)	Placebo (N=112)
Number of adverse events		*738*	*634*	*609*	*582*
Patients experiencing at least one AE		*105 (95%)*	*101 (93%)*	*100 (90%)*	*103 (92%)*
	Blood and lymphatic system disorders	3 (3%)	2 (2%)	5 (5%)	3 (3%)
	Cardiac disorders	3 (3%)	8 (7%)	1 (1%)	2 (2%)
	Ear and labyrinth disorders	3 (3%)	1 (1%)	5 (5%)	5 (4%)
	Endocrine disorders	0 (0%)	1 (1%)	0 (0%)	0 (0%)
	Eye disorders	8 (7%)	9 (8%)	13 (12%)	8 (7%)
	Gastrointestinal disorders	62 (56%)	49 (45%)	46 (41%)	36 (32%)
	General disorders and administration	28 (25%)	27 (25%)	26 (23%)	32 (29%)
	Hepatobiliary disorders	3 (3%)	0 (0%)	2 (2%)	1 (1%)
	Immune system disorders	1 (1%)	3 (3%)	1 (1%)	0 (0%)
	Infections and infestations	58 (52%)	62 (57%)	68 (61%)	69 (62%)
	Injury, poisoning and procedural complications	43 (39%)	29 (27%)	26 (23%)	28 (25%)
	Investigations	20 (18%)	17 (16%)	10 (9%)	8 (7%)
	Metabolism and nutrition disorders	9 (8%)	7 (6%)	2 (2%)	4 (4%)
	Musculoskeletal and connective tissue disorders	26 (23%)	37 (34%)	37 (33%)	29 (26%)
	Neoplasms benign, malignant and unspecified	1 (1%)	4 (4%)	2 (2%)	2 (2%)
	Nervous system disorders	46 (41%)	47 (43%)	48 (43%)	44 (39%)
	Psychiatric disorders	30 (27%)	22 (20%)	21 (19%)	22 (20%)
	Renal and urinary disorders	13 (12%)	10 (9%)	9 (8%)	5 (4%)
	Reproductive system and breast disorders	3 (3%)	2 (2%)	4 (4%)	2 (2%)
	Respiratory disorders	23 (21%)	13 (12%)	15 (14%)	16 (14%)
	Skin and subcutaneous tissue disorders	11 (10%)	13 (12%)	16 (14%)	17 (15%)
	Surgical and medical procedures	3 (3%)	8 (7%)	6 (5%)	7 (6%)
	Vascular disorders	2 (2%)	3 (3%)	4 (4%)	6 (5%)
Patients experiencing at least one SAE		*7 (6%)*	*12 (11%)*	*10 (9%)*	*13 (12%)*
	Cardiac disorders	1 (1%)	2 (2%)	0	0
	Gastrointestinal disorders	0	0	1 (1%)	1 (1%)
	General disorders and administration	2 (2%)	1 (1%)	0	1 (1%)
	Hepatobiliary disorders	1 (1%)	0	1 (1%)	1 (1%)
	Infections and infestations	1 (1%)	4 (4%)	4 (4%)	4 (4%)

	Injury, poisoning and procedural complications	0	3 (3%)	3 (3%)	2 (2%)
	Investigations	0	0	0	1 (1%)
	Musculoskeletal and connective tissue disorders	1 (1%)	0	0	0
	Neoplasms benign, malignant and unspecified	0	0	1 (1%)	0
	Nervous system disorders	0	1 (1%)	1 (1%)	0
	Psychiatric disorders	1 (1%)	1 (1%)	0	1 (1%)
	Renal and urinary disorders	1 (1%)	0	1 (1%)	0
	Respiratory disorders	0	1 (1%)	1 (1%)	0
	Skin and subcutaneous tissue disorders	0	0	0	1 (1%)
	Surgical and medical procedures	0	1 (1%)	1 (1%)	1 (1%)
	Vascular disorders	0	0	0	1 (1%)
Patients experiencing at least one SUSAR		*0 (0%)*	*1 (1%)*	*0 (0%)*	*0 (0%)*

Data are number of patients experiencing each type of event. Two patients randomised to the Riluzole treatment arm were prescribed Fluoxetine by their GP during follow-up (these were protocol deviations). These two patients experienced a total of 5 adverse events and no serious adverse events. A few of the AEs for these patients occurred after Fluoxetine was prescribed and therefore, if adverse reactions, may be attributable to Fluoxetine rather than Riluzole (or a combination of the two). Progressive change due to SPMS, in motor, sensory, balance, sphincter (including urinary tract infections), vision, cognitive and fatigue levels were not reported as AEs/SAEs/SUSARs. In addition, relapses were not reported as AEs/SAEs/SUSARs, but are collated separately. AE: adverse event, SAE: serious adverse event; SUSAR: Suspected Unexpected Serious Adverse Reaction.
